# Effects of endogenous sex hormones on lung function and symptom control in adolescents with asthma

**DOI:** 10.1186/s12890-018-0612-x

**Published:** 2018-04-10

**Authors:** Mark D. DeBoer, Brenda R. Phillips, David T. Mauger, Joe Zein, Serpil C. Erzurum, Anne M. Fitzpatrick, Benjamin M. Gaston, Ross Myers, Kristie R. Ross, James Chmiel, Min Jie Lee, John V. Fahy, Michael Peters, Ngoc P. Ly, Sally E. Wenzel, Merritt L. Fajt, Fernando Holguin, Wendy C. Moore, Stephen P. Peters, Deborah Meyers, Eugene R. Bleecker, Mario Castro, Andrea M. Coverstone, Leonard B. Bacharier, Nizar N. Jarjour, Ronald L. Sorkness, Sima Ramratnam, Anne-Marie Irani, Elliot Israel, Bruce Levy, Wanda Phipatanakul, Jonathan M. Gaffin, W. Gerald Teague

**Affiliations:** 1 University of Virginia School of Medicine, Charlottesville, VA 22908 USA; 2 Pennsylvania State University School of Medicine, Hershey, USA; 3 Lerner Research Institute, Cleveland Clinic Foundation, Cleveland, USA; 4 Emory University School of Medicine, Atlanta, USA; 5 Rainbow Babies and Children’s Hospital, Cleveland, USA; 6 San Francisco School of Medicine, University of California, San Francisco, USA; 7 University of Pittsburgh School of Medicine, Pittsburgh, USA; 8 Wake Forest University School of Medicine, Winston-Salem, USA; 9 Washington University School of Medicine, St. Louis, USA; 10 University of Wisconsin School of Medicine, Madison, USA; 11 Virginia Commonwealth University School of Medicine, Richmond, USA; 12 Harvard University School of Medicine, Boston, USA

**Keywords:** Asthma, Sex hormones, Testosterone, Estradiol, Puberty, Lung function

## Abstract

**Background:**

Although pre-puberty asthma is more prevalent in males, after puberty through middle-age, asthma is more prevalent in females. The surge of sex hormones with puberty might explain this gender switch.

**Methods:**

To examine the effects of sex hormones on lung function and symptoms with puberty, Tanner stage was assessed in 187 children 6–18 years of age (59% severe) enrolled in the NIH/NHLBI Severe Asthma Research Program (SARP). The effects of circulating sex hormones (*n* = 68; testosterone, dehydroepiandrosterone sulfate (DHEA-S), estrogen, and progesterone) on lung function and 4 week symptom control (ACQ6) in cross-section were tested by linear regression.

**Results:**

From pre−/early to late puberty, lung function did not change significantly but ACQ6 scores improved in males with severe asthma. By contrast females had lower post-BD FEV_1_% and FVC% and worse ACQ6 scores with late puberty assessed by breast development. In males log DHEA-S levels, which increased by Tanner stage, associated positively with pre- and post-BD FEV_1_%, pre-BD FVC %, and negatively (improved) with ACQ6. Patients treated with high-dose inhaled corticosteroids had similar levels of circulating DHEA-S. In females, estradiol levels increased by Tanner stage, and associated negatively with pre-BD FEV_1_% and FVC %.

**Conclusions:**

These results support beneficial effects of androgens on lung function and symptom control and weak deleterious effects of estradiol on lung function in children with asthma. Longitudinal data are necessary to confirm these cross-sectional findings and to further elucidate hormonal mechanisms informing sex differences in asthma features with puberty.

**Trial registration:**

ClinicalTrials.gov registration number: NCT01748175.

**Electronic supplementary material:**

The online version of this article (10.1186/s12890-018-0612-x) contains supplementary material, which is available to authorized users.

## Background

The prevalence of current asthma in the U.S. is higher in children than in adults, and from pre-school to age 14 years is greater in boys than it is in girls [1]. Both asthma severity and hospitalizations start to decrease in late childhood and remain relatively low into young adulthood [2, 3]. After age 18 years, in young men, current asthma prevalence is lower than in childhood; in young women although lower than in childhood, current asthma prevalence is higher than it is in young men, and remains higher in women through middle-age [1]. Surges in endogenous sex hormones across adolescence could affect the “gender switch” in asthma status [2]. For example, DHEA-S, a circulating androgen that increases with puberty, inhibits human airway smooth muscle and airway fibroblast proliferation [4, 5] and may influence airway epithelial-to-mesenchymal transition [6]. Testosterone promotes airway smooth muscle relaxation [7]. In women both with and without asthma, FEV_1_% peaks at the end of the luteal phase to the beginning of menstruation, and then decreases when circulating estrogen and progesterone decrease [8]. Accordingly, the fall in circulating estrogen and progesterone from the luteal to the follicular phase of the menstrual cycle is accompanied by a reduction in lymphocyte β_2_ adrenoceptor density [9] and increased bronchial responsiveness in women with asthma [10]. Recent studies of dysanapsis (differential growth of airway caliber and lung volume) in healthy subjects suggest that, by adulthood, women develop more small airways resistance than do men [11]. Thus these studies support interdependent effects of sex and endogenous sex hormones on lung growth, and airways responsiveness that likely inform asthma status from puberty to middle-age.

Puberty is a dynamic process regulated by hormonal signals from the central nervous system that results in sexual maturation. Assessment of the stage of pubertal maturation is different in boys than girls [12]. In boys, androgen production gradually increases both from testes producing testosterone and the adrenal glands producing weaker androgens—ultimately leading to pubarche. Girls experience increases in the production of estrogen from the ovaries (driving thelarche and ultimately menarche) and androgens such as androstenedione and DHEA-S from the adrenal glands (driving pubarche in girls) [13]. In children studies of pubertal maturation and asthma status furthermore may be affected by corticosteroid treatment [14]. The primary purpose of the current studies was to do a cross-sectional analysis of the effects of pubertal maturation and sex hormone levels on asthma features in a sample of children enrolled in SARP. We found important differences in phenotypic features according to sex and pubertal stage. In boys higher DHEA-S levels positively associated with greater FEV_1_% predicted and improved 4 week symptoms, whereas in girls, breast development, signifying estradiol effects, weakly associated with lower lung function.

## Methods

### Severe asthma research program

Details of the SARP III network, recruitment targets, enrollment and characterization procedures, and the longitudinal protocol have been previously published [15, 16]. Active tobacco smokers were excluded. Participants maintained medications for asthma as prescribed by their care provider. Study procedures were approved by the IRB at each institution and an independent Data Safety Monitoring Board (Additional file 1: Figure S1). All subjects provided written informed consent and/or assent. The study is listed on ClinicalTrials.gov.

### Criteria for enrollment

Enrollees met criteria for current asthma based on responsiveness to β-agonist (≥12% increase in FEV_1_ from baseline post-albuterol) and/or positive methacholine bronchoprovocation. Severe asthma was defined according to the ERS/ATS consensus definition [17]. Children whose asthma required treatment with high-dose inhaled corticosteroids (≥440 mcg of fluticasone equivalents per day for children 6–11 years of age; ≥880 mcg fluticasone equivalents per day ≥12 years of age) plus a second controller and/or systemic corticosteroids to maintain asthma control or which was uncontrolled despite these medications were assigned to the severe sub-group. Medications at this level were required for at least 6 of the previous 12 months and the 3 months prior to enrollment for participants to qualify for the severe sub-group. Those who did not meet criteria for severe asthma were assigned to the non-severe sub-group.

### Characterization

Characterization procedures extended from the SARP I and II protocols [18–20] for SARP III included detailed history and physical examination, vital signs, height, weight, characterization questionnaires, assessment of four-week symptom control (asthma control questionnaire; ACQ6 [21]), and spirometry with maximal β-agonist reversibility testing (dose escalation protocol at 180 mcg albuterol increments up to 720 mcg).

Blood was drawn during the visit but not with systematic timing related to the timing of the spirometry. Pubertal stage was determined by study coordinators and investigators certified a priori to enrollment by a pediatric endocrinologist (MDD) with the method of Marshall and Tanner [12]. To identify central (CNS) puberty, Tanner stage for males was based on pubic hair and for females breast development. Females were also separated into pre−/early adrenarche and mid−/late adrenarche based on Tanner stages of pubic hair.

Spirometry, maximum bronchodilator challenge and methacholine challenge were performed by study coordinators certified after standard training and completion of an examination administered by the SARP III Data Coordinating Center on ATS-compatible equipment. Quality analysis of lung function tests and validation of results was accomplished by inspection of randomly sampled flow volume plots by a central over-reader. Population reference standards adjusted for age, height, and sex were from the Global Lung Initiative (GLI) [22].

### Sex hormone assays

Sex hormone and SHBG levels were analyzed at the University of Virginia Center for Research in the Reproduction Ligand Core Laboratory. Estradiol levels were assessed with Calbiotech ELISA and all other metabolites were assessed using the Siemens Immulite 2000. Free testosterone was calculated from total testosterone and SHBG as previously described [23]. The lower limit of detection for assays were: testosterone 20 ng/dL; DHEAS 15 μg/dL; progesterone 0.20 ng/mL; and SHBG 10 nmol/L. For the purpose of mathematical analyses, individuals with measurements below the limit of detection were counted as having a value just below the lower limit of detection. Intra−/inter-assay coefficients of variation (CVs, %) were as follows: estradiol 6.3/8.1, testosterone 4.4/7.5, DHEAS 5.4/6.5, progesterone 4.4/7.8, and SHBG 2.7/5.2.

### Data analysis

The analysis was restricted to enrollees with complete data sets with regards to Tanner stage and lung function. Because of natural variation in recruitment, mid-Tanner stages were relatively under-represented (Table 1). To examine the effects of pubertal maturation, participants were assigned to sub-groups including those with pre−/early puberty (Tanner 1–2) and mid-late puberty (Tanner 3–5).

**Table 1:** SARP III Pediatric sample features compared by sex

	Males (*n* = 116)	Females (*n* = 71)		*P* value
Age, years, mean (± sd)	11.3 (± 2.7)	11.8 (± 3.1)		0.21
Age of asthma diagnosis	3.1 (± 2.7)	3.1 (± 2.7)		0.90
Years since onset of asthma symptoms (asthma duration)	9.0 (± 3.2)	9.4(± 3.5)		0.50
BMI percentile	76.9 (± 26.5)	73.5 (± 27.1)		0.40
Race/ethnicity, n (%)				
White non-Hispanic	28 (24)	26 (37)		0.26
African American non-Hispanic	51 (44)	26 (37)		
Hispanic	17 (15)	11 (15)		
Other	20 (17)	8 (11)		
Asthma severity, n (%)				
Severe	66 (57)	44 (62)		0.49
Not severe	50 (43)	27 (38)		
Systemic Corticosteroid Treatment (current)	11 (9)	5 (7)		0.56
Systemic Corticosteroid Treatment (past 3 mos)	7 (6)	3 (4)		0.59
High-Dose Inhaled Corticosteroids	82 (71)	50 (70)		0.97
Low-Medium-Dose Inhaled Corticosteroids	23 (20)	14 (20)		0.99
Montelukast (during last 12 months) n(%)	71 (60)	54 (76)		0.03
Long-acting beta agonist	86 (74)	49 (69)		0.45
Atopic sensitization				
At Least One Positive Specific IgE (of 15 tests)	106 (93)	64 (91)		0.70
Number of Positive Specific IgEs (of 15 tests). Mean (± sd)	7.0 (4.3)	7.0 (4.9)		0.95
History of atopic dermatitis. n(%)	74 (64)	44 (62)		0.80
Tanner stage, n (%)	Males	Females		
	Pubic hair	Breast development	Pubic hair	
I	67 (58)	14 (20)	24 (34)	
II	17 (15)	18 (25)	13 (18)	
III	8 (7)	16 (23)	8 (11)	
IV	7 (6)	9 (13)	13 (18)	
V	17 (15)	14 (20)	13 (18)	

Categorical data are presented as count and percentage, and continuous data as means ± standard deviations. Comparisons of differences by pubertal stage between males and females (both adrenarche and breast development) was performed by testing the stage by sex interaction term in a simple regression model. PC_20_ was evaluated on a log base 2 scale, with two zero values replaced by 0.016, or half of the minimum non-zero value. Univariable linear regression was used to evaluate relationships between measures of lung function or ACQ6 and individual 5-level Tanner stage. Multivariable linear regression tested associations of clinical outcomes (lung function and ACQ6) with sex hormone levels (for males, testosterone and DHEA-S; for females, estradiol, progesterone, free testosterone and DHEAS) as co-variates. With a backward selection method, hormones that were not significantly associated with the outcome were removed in steps and only significant associations were reported. These analyses were then further stratified based on BMI ≥85th percentile and < 85th percentile. *P*-values are unadjusted in these exploratory post hoc analyses.

## Results

### Sample features

The sample included 116 males and 71 females (Table 1). There were no significant differences between males and females with regard to age, racial/ ethnic background, asthma severity, or corticosteroid treatment. There were 110 severe and 77 non-severe participants in the analytic cohort. Of these, 74 severe- and 30 non-severe participants had an ACQ < 1.0 as an estimate of poor control (67% and 39%, respectively) [24]. Overall the sample was enriched in African Americans and severe asthma, with a high proportion with atopic sensitization. Among males, 84 (63%) were in pre−/early puberty and 32 (28%) in mid−/late puberty by Tanner stages of pubic hair development. Among females, 32 (45%) were in pre−/early puberty and 39 (56%) in mid−/late puberty by Tanner stages of breast development. 37 females (52%) were in pre−/early- and 34 (47%) in mid−/late adrenarche by Tanner stages of pubic hair. Adolescent females discordant for breast and pubic hair stages of development included 6 wherein pubic hair stage exceeded breast stage, and 1 wherein breast stage exceeded pubic hair (Additional file 1: Table S1).

### Differences in sex hormones with puberty

68 children in the sample had circulating sex hormones measured (45 males, 23 females). From pre−/early to mid−/late puberty, testosterone increased in males, and estradiol increased in females (Additional file 1: Figure S2A and B). Likewise with puberty DHEA-S increased in males and DHEA-S, progesterone, and free testosterone increased in females (results not shown).

### Differences in lung function with puberty

For males, there was a tendency for lung function and the maximum bronchodilator response to improve with pubertal maturation, but these differences were not statistically significant (Table 2). By contrast in females, both the post-bronchodilator FEV_1_% and FVC % were significantly lower (by 8.9% and 9.1% respectively, *p* = 0.01 each) from pre−/early to mid−/late puberty as determined by breast development. In an analysis limited to severe females alone (*n* = 44), the lower FEV_1_ (%) from early to late puberty remained significant. Similarly, the differences by pubertal maturation remained when participants were stratified by overweight/obesity status (Additional file 1: Table S2-A-1 and S2-A-2). These differences by Tanner stage are attributable to breast and not pubic hair development, supporting a likely estradiol effect.

**Table 2:** Lung function by sex and pubertal stage in children 6–18 years with asthma

	Males				Females				Females			
	Tanner stages of pubic hair†				Tanner stages of breast development†				Tanner stages of pubic hair†			
	1–2 *n* = 84	3–5 *n* = 32	Ratio 3–5:1–2	∆ 3–5:1–2	1–2 *n* = 32	3–5 *n* = 39	Ratio 3–5:1–2	∆ 3–5:1–2	1–2 *n* = 37	3–5 *n* = 34	Ratio 3–5:1–2	∆ 3–5:1–2
Pre-BD FEV1 (%)	89.4 ± 18.5	89.1 ± 16.4	1.00	− 0.3	93.9 ± 13.0	87.2 ± 14.9	0.93	−6.8	90.4 ± 15.2	90.1 ± 13.7	1.00	− 0.3
Max Post-BD FEV1 (%)	103.5 ± 19.3	105.7 ± 10.2	1.02	2.2	109.4 ± 13.1	100.5 ± 13.1	0.92	− 8.9*	106.0 ± 14.4	102.9 ± 13.0	0.97	− 3.1
BD Resp. (% FEV_1_ change)	17.3 ± 16.6	21.8 ± 20.8	1.26	4.5	17.2 ± 10.7	17.2 ± 17.5	1.00	−0.0	19.1 ± 17.1	15.2 ± 11.5	0.80	−3.9
Absolute BD Resp. (net FEV_1_ change)	14.1 ± 10.3	16.7 ± 11.7	1.18	2.5	15.5 ± 8.3	13.4 ± 9.2	0.86	−2.1	15.7 ± 9.7	12.9 ± 7.6	0.82	−2.8
Pre-BD FVC (%)	101.4 ± 15.9	106.4 ± 16.0	1.05	5.0	106.3 ± 11.6	100.5 ± 12.9	0.94	−5.9*	102.9 ± 13.5	103.3 ± 11.8	1.01	0.4
Max Post-BD FVC (%)	109.2 ± 17.6	112.5 ± 13.8	1.03	3.3	113.8 ± 12.9	104.8 ± 13.7	0.92	−9.0*	110.4 ± 14.9	107.2 ± 13.0	0.97	− 3.2
Pre-BD FEV1/FVC (%)	87.3 ± 10.7	83.5 ± 11.6	0.96	−3.8	87.9 ± 8.4	86.3 ± 10.9	0.98	−1.5	87.2 ± 9.9	86.8 ± 9.9	1.00	−0.4
Max Post-BD FEV1/FVC (%)	94.9 ± 8.8	94.9 ± 7.4	1.00	0.0	97.2 ± 7.6	96.4 ± 8.2	0.99	−0.8	97.1 ± 7.8	96.4 ± 8.1	0.99	− 0.6
PC20 (log 2)	0.3 ± 1.9 *n* = 35	1.0 ± 3.0 *n* = 10	3.57	0.7	−0.7 ± 3.6 *n* = 7	0.0 ± 2.4 *n* = 15	− 0.05	0.7	− 0.3 ± 3.3 *n* = 10	−0.1 ± 2.4 *n* = 12	0.47	0.2

Ϯ Cross sectional, based on enrollment examination; * p < 0.05 Tanner Stage 1–2 versus Tanner Stage 3–5

### Differences in ACQ6 with puberty

With regards to four week symptom control (Table 3), with males (*n* = 116) the ACQ6 was not significantly different from pre−/early to mid−/late puberty, whereas in females (*n* = 71) the ACQ6 was higher (worse) with puberty assessed by breast bud (*p* < 0.05) and by pubic hair development (*p* < 0.05). In males with severe but not non-severe asthma, the ACQ6 was lower (improved) with pubertal maturation, whereas the ACQ6 was higher with pubertal stage in females with both severe (*n* = 44) and non-severe (*n* = 37) asthma (*p* < 0.05 for both). Again, the differences by pubertal maturation remained when participants were stratified by overweight/obesity status (Additional file 1: Table S2-B-1 and S2-B-2).

**Table 3:** ACQ6 by sex, pubertal stage, and asthma severity in children 6–18 years of age

	Males				Females				Females			
	Tanner stages of pubic hair†				Tanner stages of breast development†				Tanner stages of pubic hair†			
	1–2 *n* = 84	3–5 *n* = 32	Ratio 3–5:1–2	∆ 3–5:1–2	1–2 *n* = 32	3–5 *n* = 39	Ratio 3–5:1–2	∆ 3–5:1–2	1–2 *n* = 37	3–5 *n* = 34	Ratio 3–5:1–2	∆ 3–5:1–2
ACQ6	1.2 ± 0.9	0.8 ± 0.7	0.70	−0.4	1.0 ± 0.8 *n* = 32	1.3 ± 1.0 *n* = 39	1.30	0.3*	1.1 ± 0.9 *n* = 37	1.3 ± 1.0 *n* = 34	1.24	0.3*
Severe												
ACQ6	1.5 ± 0.9 *n* = 49	0.9 ± 0.6 *n* = 17	0.63	−0.6*	1.1 ± 1.0 *n* = 18	1.3 ± 1.0 *n* = 26	1.18	0.2*	1.1 ± 1.0 *n* = 21	1.4 ±1.0 *n* = 23	1.22	0.3*
Non-severe												
ACQ6	0.8 ± 0.7 *n* = 35	0.7 ± 0.9 *n* = 15	0.94	−0.0	0.9 (0.6), *n* = 14	1.4 (1.1), *n* = 13	1.50	0.5*	1.0 (0.7), *n* = 16	1.3 (1.0), *n* = 11	1.25	0.3*

*p < 0.05, Tanner Stage I-II compared to Tanner Stage III-V

### Multi-variable linear regression of lung function and ACQ6 with sex hormones

#### Males

For males in the sample 6–18 years of age, lung function associated positively with log DHEA-S values (Table 4). The pre-bronchodilator FEV_1_% (β = 8.05; *p* = 0.01), post-BD FEV_1_% (β = 8.82, *p* = 0.008), and pre-BD FVC % (β = 8.33, *p* = 0.01) had strongly positive β coefficients. The ACQ6 had a negative β coefficient with higher log DHEA-S values (β = − 0.59, *p* = 0.007), indicating 4 week symptom improvement. These associations were driven largely by participant who were overweight/obese (Additional file 1: Table S3-A-1 and S3-A-2). The final model predicting ACQ6 included negative β coefficients for both log DHEA-S and testosterone, as well as the interaction of log DHEA-S with testosterone, an association not present when the cohort was stratified by overweight/obesity status. Circulating DHEA-S levels in males did not vary significantly when compared according to treatment with high-dose versus medium-dose inhaled corticosteroids (Additional file 1: Figure S3).

**Table 4:** Linear regression analysis of lung function and ACQ6 by sex hormone levels in males 6–18 years with asthma

Outcome variable	Covariate remaining in the model*	Beta coefficient	Covariate *p* value	R squared	*P* value of the model
Pre-BD FEV_1_ (%)	Log (DHEA-S)	9.285	0.011	0.168	0.011
Post-BD FEV_1_ (%)	Log (DHEA-S)	9.527	0.006	0.160	0.006
Pre-BD FVC (%)	log (DHEA-S)	9.121	0.007	0.157	0.007
ACQ6	Log (DHEA-S)	−0.008	0.019	0.214	0.018
	Testosterone	−0.005	0.031		
	log (DHEA-S) *Testosterone #	0.003	0.05		

*Results reflect final results of a model that started with testosterone and DHEA-S. Covariates that were not significantly associated with the outcome in this model were then removed by backward selection and model was re-run with remaining variables. # A log (DHEA-S) * testosterone interaction was found and included in the final model

#### Females

For females 6–18 years of age, androgens associated positively, but estrogens negatively with lung function (Table 5). Free testosterone had a positive association with post-BD FEV_1_% (β = 3.99, *p* = 0.04). In contrast, estradiol had negative β coefficients for pre-BD FEV_1_% (β = − 0.47, *p* = 0.03), post-BD FEV_1_% (β = − 0.62, *p* = 0.0009), pre-BD FVC % (β = − 0.36, *p* = 0.04), and post-BD FVC % (β = − 0.33, *p* = 0.03). These associations were not significant in either group when stratified by overweight/obesity status (Additional file 1: Table S3-B-1 and S3-B-2). For the ACQ6, only log DHEA-S had a significant association, it varied positively (β = 0.49, *p* = 0.05) and the correlation was relatively weak. ACQ6 was not associated with age (Additional file 1: Table S4).

**Table 5:** Multi-variable regression analysis of lung function and ACQ6 by sex hormone levels in females 6–18 years with asthma

Outcome variable	Covariate remaining in the model*	Beta coefficient	Covariate p value	R squared	*P* value of the model
Pre-BD FEV_1_ (%)	Estradiol	−0.457	0.028	0.209	0.028
Post-BD FEV_1_ (%)	Estradiol	−0.454	0.005	0.320	0.005
Pre-BD FVC (%)	Estradiol	−0.351	0.025	0.217	0.025
Post-BD FVC (%)	Estradiol	−0.322	0.015	0.249	0.015
ACQ6	log (DHEA-S)	0.006	0.048	0.173	0.048

*Results reflect final results of a model that started with log (DHEA-S), estradiol, progesterone, and free testosterone as covariates. Covariates that were not significantly associated with the outcome in this model were then removed by backward selection and model was re-run with remaining variables

## Discussion

In children enriched in severe asthma enrolled in the NIH/NHLBI SARP III study (*n* = 187), there were important differences in lung function and four-week symptom control between males and females with pubertal maturation. In males, lung function was not different and symptom control improved when comparing those in early vs. late puberty, whereas in females lung function and symptom control were worse. Circulating sex hormones changed with puberty; in males greater androgen levels associated with better lung function and ACQ6, while in females greater circulating estrogens had a weak but significant negative association with lung function. These results support the general hypothesis that with pubertal maturation asthma severity is likely to improve in males but worsen in females [2]. Furthermore our data suggest a beneficial effect of endogenous androgens on lung function growth and ACQ6 in males in later puberty; and that endogenous estrogens may have weak deleterious effects on lung function growth in girls.

A beneficial effect of androgens is suggested by the presence of fewer asthma symptoms in late Tanner stage males with severe asthma. In females the presence of more asthma symptoms in late adolescence either reinforces the benefit of androgens (given an absence of significant androgens in females) or supports a negative role for estradiol. These integrated, Tanner-stage-based measurements of the effect of sex hormones over time are complemented by the association between DHEA-S and fewer asthma symptoms (lower ACQ6) and higher pre- and post-albuterol FEV1% observed in boys. Given that GLI-based normal ranges of lung function are age-specific these maturational and hormone-associated measures appear to be present beyond the expected changes in lung function based on age alone. Furthermore, we did not note associations between ACQ6 and age in either boys or girls. Note that it is unlikely that the level of corticosteroid therapy caused the male/female discordance we observed because the males and females did not differ with regard to oral or inhaled corticosteroid dosing (Table 1). Furthermore treatment with high-dose corticosteroids did not suppress DHEA-S levels in males. Although the testosterone increase is quantitatively orders of magnitude greater than that of DHEA-S with puberty [25, 26], DHEA-S levels in our analysis were more strongly associated with better lung function and improved ACQ6 than was testosterone.

Of note, androgen receptors are expressed in bronchial smooth muscle tissue, and testosterone relaxes contracted airway smooth muscle, suggesting a beneficial effect of androgens on airway smooth muscle tone [7]. Additionally, DHEA-S inhibits human airway smooth muscle and airway fibroblast proliferation [5], and may influence airway epithelial-to-mesenchymal transition [6], suggesting that androgens may prevent adverse airway remodeling seen in severe asthma. Together, these affects could be associated with the lower asthma symptom scores we observed in association with increased DHEA-S and with late adolescence. Indeed a trial of androgen administration provided early data regarding safety and improved ACQ [27]. We noted associations between DHEA-S and lung function that were present in overweight/obese but not normal weight. This differential effect by weight status, which was seen only in boys, is of unclear significance and requires further investigation.

In females, subtle but significant discordance between loss of lung function observed in association with late puberty assessed by breast staging relative to pubic hair staging suggests that estrogen, which drives breast development, may be the main factor affecting lung function. Only females have a surge of estrogen or progesterone during puberty, and breast development is exclusively associated with this surge [28]. In our study the number of females with discordant pubertal examinations was small, whereas adrenal androgen effects confounded all assessments. We also cannot exclude an adverse effect of progesterone. For example, the effects of pregnancy on lung function may be either both estrogen and progesterone-dependent, and both hormones have been hypothesized to play roles in the worsening of lung function [2].

These results likewise support disparate changes in lung growth between males and females with puberty that bear on the remarkable decline in hospitalizations and asthma severity seen in young adults [3]. Asthma prevalence appears to decline more in males than it does in females by the second decade of life [1], a result which might be explained by disparate trends in airway growth due to sex hormones. Young adult females exhibit a “dysanaptic” pattern of airway growth which supports a pattern of anatomically “fixed” airflow limitation [11]. With regards to airway hyperreactivity, we found that the level of methacholine responsiveness actually fell in both males and females with Tanner stage, a finding differs from the results of Tantisera et al. [29] wherein females had increased methacholine responsiveness. The difference between the two studies may be due to sample differences, wherein the SARP III cohort is relatively enriched with African Americans and children with severe asthma.

It should also be noted that other hormonal changes during puberty could also affect lung function, including an increase in the degree of insulin resistance [30]—which itself is associated with increased risk of asthma in adults [31] and adolescent girls [32]—and elevations in stress hormones, including cortisol [33]—with such stress systems further associated with asthma risk [34]. While temporally related (i.e., during puberty), the processes of insulin resistance and stress response go beyond sex hormone regulation alone [30, 33]. Unfortunately, the timing of our blood collection was not specifically in the early morning (thus not as helpful to assess cortisol regulation) or with fasting status (for assessing insulin resistance), and thus we were not able to assess how these processes may have interacted with changes in sex hormones. Nevertheless, because we were able to assess relationships between measured levels of sex steroids and outcomes important to asthma severity, our findings suggest a direct role for sex steroids themselves.

This cross-sectional analysis of the effects of puberty on asthma features is exploratory and intended primarily to identify specific hypotheses to be tested with longitudinal measurements. We would point out that the veracity of the results is limited by the cross-sectional analytic approach, and the relative paucity of children who were in the mid-Tanner stages (III/IV) at the time of enrollment and our lack of knowledge for most female participants of whether central puberty or adrenarche began first. We lacked data on some participant characteristics, including depression status—which could affect stress response [34]—and the fraction of excreted nitrosoxide (FeNO)—which would have permitted assessment of the relationship between hormone levels with activity of Th2-high airway inflammation.

## Conclusions

These results impact understanding of the differences in asthma features with pubertal maturation and the potential for new treatment. The androgen surge with puberty is likely to confer protective effects on lung growth in both males and females whereas estrogens may well have deleterious effects in females extending into adult development. A trial of weak androgen therapy in adolescent children with low androgen levels and poorly-controlled asthma could be considered to improve lung function. These data may help to explain the gender switch observed in asthma incidence during adolescence and likewise inform lung growth and asthma severity with subsequent maturation into adulthood.

## Additional file

Additional file 1: Sex Hormones & Asthma. Type of data: Tables and Figures. (DOCX 468 kb)

## Abbreviations

ACQ6: Asthma control questionnaire 6 item scale
DHEA-S: Dehydroepiandrostenedione sulfate
FEV_1_%: Forced expired volume in one second percent predicted
FVC %: Forced expired vital capacity % predicted
NIH/NHLBI: National Institutes of Health/National Heart Lung Blood Institute
SARP: Severe Asthma Research Program
