# Supplementary material for: Effects of endogenous sex hormones on lung function and symptom control in adolescents with asthma
Source: BMC Pulm Med. 2018 Apr 10;18:58. doi: 10.1186/s12890-018-0612-x (PMC5891903; doi:10.1186/s12890-018-0612-x)
Supplement: Supplementary file 1 — Sex Hormones & Asthma. Type of data: Tables and Figures. (DOCX 468 kb) [file 12890_2018_612_MOESM1_ESM.docx]

**On-line Data Supplement**

**Effects of Endogenous Sex Hormones on Symptom Control and Lung Function in Adolescents with Asthma**

Mark D. DeBoer, MD ^1^, Brenda R. Phillips, MS ^2^, David T. Mauger, PhD ^2^, Joe Zein, MD ^3^, Serpil C. Erzurum, MD ^3^, Anne M. Fitzpatrick, PhD ^4^, Benjamin M. Gaston, MD ^5^, Ross Myers, MD ^5^, Kristie R. Ross, MD ^5^, James Chmiel, MD, MPH ^5^, Min Jie Lee, MD ^5^, John V. Fahy, MD, MSc ^6^, Michael Peters, MD ^6^, Ngoc P. Ly, MD, MPH ^6^, Sally E. Wenzel, MD ^7^, Merritt L. Fajt, MD ^7^, Fernando Holguin, MD ^7^, Allyson Larkin, MD ^7^, Wendy C. Moore, MD ^8^, Stephen P. Peters, MD, PhD ^8^, Mario Castro, MD ^9^, MPH, Andrea M. Coverstone, MD ^9^, Leonard B. Bacharier MD ^9^, Nizar N. Jarjour, MD ^10^, Ronald L. Sorkness, PhD ^10^, Sima Ramratnam, MD ^10^, Anne-Marie Irani, MD ^11^, Elliot Israel, MD ^12^, Bruce Levy, MD ^12^, Wanda Phipatanakul MD, MS ^12^, Jonathan M. Gaffin, MD, MMSc ^12^, and W. Gerald Teague, MD ^1^

**Affiliations**

^1^ University of Virginia School of Medicine; ^2^ Pennsylvania State University School of Medicine; ^3^ Lerner Research Institute, Cleveland Clinic Foundation; ^4^ Rainbow Babies and Children’s Hospital; ^5^ University of California, San Francisco School of Medicine; ^6^ University of Pittsburgh School of Medicine; ^7^ Emory University School of Medicine; ^8^ Wake Forest University School of Medicine; ^9^ Washington University School of Medicine; ^10^ University of Wisconsin School of Medicine; ^11^ Virginia Commonwealth University School of Medicine; ^12^ Harvard University School of Medicine.

**Corresponding Author**

W. Gerald Teague, MD

Ivy Foundation Distinguished Professor of Pediatrics

University of Virginia School of Medicine

Charlottesville, VA 22908

Email: [wgt2p@virginia.edu](mailto:wgt2p@virginia.edu)/Phone: 434-243-06

**On-line Supplement Tables**

| **Table S1**  **Characteristics of Females Discordant for Breast and Pubic Hair Stages of Pubertal Development** | | | |
| --- | --- | --- | --- |
|  | Breast development > pubic hair  (n=1) | Pubic hair > breast development (n=6) | No Discrepancy |
| Age, mean ± sd | 9.3 | 12.1 ± 0.8 | 11.9 ± 3.23 |
| Race/ethnicity, n (%) |  |  |  |
| White non-Hispanic | 0 (0) | 2 (33) | 24 (38) |
| African American non-Hispanic | 1 (100) | 3 (50) | 22 (34) |
| Hispanic | 0 (0) | 1 (17) | 10 (16) |
| Other | 0 (0) | 0 (0) | 8 (13) |
| Asthma severity, n (%) |  |  |  |
| Severe | 1 (100) | 3 (50) | 23 (36) |
| Not severe | 0 (0) | 3 (50) | 41 (64) |

| **Table S2-A-1**  **Lung Function by Sex and Pubertal Stage in Children 6-18 Years with Asthma**  **BMI ≥ 85%** | | | | | | | | | | | | | | |
| --- | --- | --- | --- | --- | --- | --- | --- | --- | --- | --- | --- | --- | --- | --- |
|  | Males | | | |  | Females | | | |  | Females | | | |
|  | Tanner stages of pubic hair† | | | |  | Tanner stages of breast development† | | | |  | Tanner stages of pubic hair† | | | |
|  | 1-2  n=49 | 3-5  n=18 | Ratio  3-5:  1-2 | ∆  3-5:  1-2 |  | 1-2  n=16 | 3-5  n=21 | Ratio  3-5:  1-2 | ∆  3-5:  1-2 |  | 1-2  n=17 | 3-5  n=20 | Ratio  3-5:  1-2 | ∆  3-5:  1-2 |
| Pre-BD FEV1  (%) | 91.6 (20.5) | 89.0 (19.7) | 0.97 | -2.6 |  | 92.6 (12.1) | 90.0 (13.0) | 0.97 | -2.6 |  | 92.5 (11.8) | 89.9 (13.4) | 0.97 | -2.5 |
| Max Post-BD FEV1  (%) | 106.5 (21.4) | 106.7 (10.5) | 1.00 | 0.3 |  | 110.7 (11.7) | 102.8 (12.5) | 0.93 | -7.9* |  | 110.2 (11.5) | 102.9 (12.8) | 0.93 | -7.4* |
| BD Resp.  (% FEV_­1_­ change) | 17.9 (16.9) | 24.2 (23.4) | 1.36 | 6.4 |  | 20.3 (9.4) | 14.9 (8.7) | 0.74 | -5.4 |  | 19.9 (9.3) | 15.1 (8.9) | 0.76 | -4.8 |
| Absolute BD Resp.  (net FEV_­1_ change) | 14.9 (10.7) | 17.7 (11.7) | 1.19 | 2.9 |  | 18.1 (6.9) | 12.9 (6.4) | 0.71 | -5.3 |  | 17.7 (6.9) | 12.9 (6.6) | 0.73 | -4.8 |
| Pre-BD FVC  (%) | 104.2 (17.3) | 108.2 (18.3) | 1.04 | 4.0 |  | 106.4 (12.2) | 103.8 (11.9) | 0.98 | -2.6 |  | 105.8 (12.1) | 104.2 (12.1) | 0.99 | -1.6 |
| Max Post-BD FVC (% ) | 112.4 (19.1) | 114.8 (13.7) | 1.02 | 2.4 |  | 116.6 (13.4) | 108.4 (13.0) | 0.93 | -8.2 |  | 115.6 (13.6) | 108.8 (13.2) | 0.94 | -6.8 |
| Pre-BD FEV1/  FVC (%) | 86.6 (11.2) | 81.7 (11.5) | 0.94 | -5.0 |  | 86.6 (8.5) | 86.3 (8.3) | 1.00 | -0.3 |  | 87.1 (8.5) | 85.9 (8.3) | 0.99 | -1.2 |
| Max Post-BD FEV1/FVC (%) | 94.3 (8.9) | 93.6 (6.8) | 0.99 | -0.7 |  | 96.1 (7.3) | 95.2 (7.0) | 0.99 | -0.9 |  | 96.4 (7.2) | 94.9 (7.0) | 0.98 | -1.5 |
| PC20 (log 2) | 0.7 (1.6), n=18 | 1.2 (3.3), n=7 | 1.70 | 0.5 |  | 1.6 (0.9) , n=2 | -0.5 (2.8) , n=7 | -.32 | -2.1 |  | 1.6 (0.9) , n=2 | -0.5 (2.8) , n=7 | -.32 | -2.1 |
| Ϯ Cross sectional, based on enrollment examination; * p < 0.05 Tanner Stage 1-2 versus Tanner Stage 3-5. | | | | | | | | | | | | | | |

| **Table S2-A-2**  **Lung Function by Sex and Pubertal Stage in Children 6-18 Years with Asthma**  **BMI < 85%** | | | | | | | | | | | | | | |
| --- | --- | --- | --- | --- | --- | --- | --- | --- | --- | --- | --- | --- | --- | --- |
|  | Males | | | |  | Females | | | |  | Females | | | |
|  | Tanner stages of pubic hair† | | | |  | Tanner stages of breast development† | | | |  | Tanner stages of pubic hair† | | | |
|  | 1-2  n=35 | 3-5  n=14 | Ratio  3-5:  1-2 | ∆  3-5:  1-2 |  | 1-2  n=16 | 3-5  n=18 | Ratio  3-5:  1-2 | ∆  3-5:  1-2 |  | 1-2  n=37 | 3-5  n=34 | Ratio  3-5:  1-2 | ∆  3-5:  1-2 |
| Pre-BD FEV1  (%) | 86.3 (15.1) | 89.2 (11.7) | 1.03 | 2.9 |  | 95.3 (14.2) | 83.9 (16.7) | 0.88 | -11.4* |  | 88.6 (17.8) | 90.2 (14.8) | 1.02 | 1.7 |
| Max Post-BD FEV1  (%) | 99.3 (15.5) | 104.4 (10.1) | 1.05 | 5.1 |  | 108.1 (14.7) | 97.8 (13.7) | 0.90 | -10.3* |  | 102.5 (15.9) | 103.0 (13.9) | 1.01 | 0.5 |
| BD Resp.  (% FEV_­1_­ change) | 16.5 (16.3) | 18.6 (17.1) | 1.13 | 2.2 |  | 14.1 (11.3) | 19.9 (24.1) | 1.41 | 5.8 |  | 18.5 (21.9) | 15.4 (14.9) | 0.83 | -3.1 |
| Absolute BD Resp.  (net FEV_­1_ change) | 13.0 (9.8) | 15.3 (12.0) | 1.17 | 2.2 |  | 12.8 (8.9) | 14.0 (11.8) | 1.09 | 1.2 |  | 13.9 (11.5) | 12.7 (9.1) | 0.92 | -1.2 |
| Pre-BD FVC  (%) | 97.4 (12.9) | 104.2 (12.9) | 1.07 | 6.8 |  | 106.2 (11.3) | 96.6 (13.3) | 0.91 | -9.7* |  | 100.5 (14.4) | 102.1 (11.6) | 1.02 | 1.6 |
| Max Post-BD FVC (% ) | 104.8 (14.4) | 109.6 (14.0) | 1.05 | 4.8 |  | 111.1 (12.2) | 100.7 (13.6) | 0.91 | -10.4* |  | 106.0 (14.8) | 105.0 (12.8) | 0.99 | -1.0 |
| Pre-BD FEV1/  FVC (%) | 88.2 (10.2) | 85.9 (11.8) | 0.97 | -2.4 |  | 89.1 (8.4) | 86.4 (13.7) | 0.97 | -2.7 |  | 87.4 (11.2) | 88.1 (12.2) | 1.01 | 0.7 |
| Max Post-BD FEV1/FVC (%) | 95.9 (8.6) | 96.6 (8.1) | 1.01 | 0.8 |  | 98.3 (8.0) | 97.8 (9.4) | 1.00 | -0.4 |  | 97.6 (8.4) | 98.7 (9.3) | 1.01 | 1.0 |
| PC20 (log 2) | -0.2 (2.0) , n=17 | 0.5 (3.0) , n=3 | -3.2 | 0.7 |  | -1.6 (4.0) , n=5 | 0.5 (2.0) , n=8 | -.31 | 2.2 |  | -0.8 (3.6) , n=8 | 0.4 (1.9) , n=5 | -.53 | 1.2 |
| Ϯ Cross sectional, based on enrollment examination; * p < 0.05 Tanner Stage 1-2 versus Tanner Stage 3-5. | | | | | | | | | | | | | | |

| **Table S2-B-1**  **ACQ6 by Sex, Pubertal Stage, and**  **Asthma Severity in Children 6-18 Years of Age**  **BMI ≥ 85%** | | | | | | | | | | | | | | |
| --- | --- | --- | --- | --- | --- | --- | --- | --- | --- | --- | --- | --- | --- | --- |
|  | Males | | | |  | Females | | | |  | Females | | | |
|  | Tanner stages of pubic hair† | | | |  | Tanner stages of breast development† | | | |  | Tanner stages of pubic hair† | | | |
|  | 1-2  n=49 | 3-5  n=18 | Ratio  3-5:  1-2 | ∆  3-5:  1-2 |  | 1-2  n=16 | 3-5  n=21 | Ratio  3-5:  1-2 | ∆  3-5:  1-2 |  | 1-2  n=17 | 3-5  n=20 | Ratio  3-5:  1-2 | ∆  3-5:  1-2 |
| ACQ6 | 1.2 (0.9) | 1.0 (0.9) | 0.84 | -0.2 |  | 1.3 (1.0) | 1.6 (1.1) | 1.27 | 0.3 |  | 1.3 (1.0) | 1.6 (1.1) | 1.25 | 0.3 |
| **Severe** | | | | | | | | | | | | | | |
| ACQ6 | 1.3 (1.0) , n=32 | 0.9 (0.6) , n=10 | 0.69 | -0.4 |  | 1.5 (1.1) , n=11 | 1.7 (1.1) , n=14 | 1.13 | 0.2 |  | 1.5 (1.1) , n=11 | 1.7 (1.1) , n=14 | 1.13 | 0.2 |
| **Non-severe** | | | | | | | | | | | | | | |
| ACQ6 | 0.8 (0.6) , n=17 | 1.0 (1.2) , n=8 | 1.27 | 0.2 |  | 0.8 (0.6) , n=5 | 1.5 (1.3) , n=7 | 1.87 | 0.7 |  | 0.9 (0.7) , n=6 | 1.5 (1.4) , n=6 | 1.56 | 0.5 |
| *p < 0.05, Tanner Stage I-II compared to Tanner Stage III-V. | | | | | | | | | | | | | | |

| **Table S2-B-2**  **ACQ6 by Sex, Pubertal Stage, and**  **Asthma Severity in Children 6-18 Years of Age**  **BMI < 85%** | | | | | | | | | | | | | | |
| --- | --- | --- | --- | --- | --- | --- | --- | --- | --- | --- | --- | --- | --- | --- |
|  | Males | | | |  | Females | | | |  | Females | | | |
|  | Tanner stages of pubic hair† | | | |  | Tanner stages of breast development† | | | |  | Tanner stages of pubic hair† | | | |
|  | 1-2  n=35 | 3-5  n=14 | Ratio  3-5:  1-2 | ∆  3-5:  1-2 |  | 1-2  n=16 | 3-5  n=18 | Ratio  3-5:  1-2 | ∆  3-5:  1-2 |  | 1-2  n=20 | 3-5  n=14 | Ratio  3-5:  1-2 | ∆  3-5:  1-2 |
| ACQ6 | 1.2 (1.0) | 0.7 (0.5) | 0.54 | -0.6* |  | 0.8 (0.6) | 1.0 (0.8) | 1.29 | 0.2 |  | 0.9 (0.8) | 1.0 (0.7) | 1.07 | 0.1 |
| **Severe** | | | | | | | | | | | | | | |
| ACQ6 | 1.7 (0.8) , n=17 | 0.9 (0.6) , n=7 | 0.53 | -0.8* |  | 0.7 (0.6) , n=8 | 1.0 (0.8) , n=11 | 1.50 | 0.3 |  | 0.8 (0.7) , n=11 | 1.0 (0.8) , n=8 | 1.30 | 0.2 |
| **Non-severe** | | | | | | | | | | | | | | |
| ACQ6 | 0.8 (0.9) , n=18 | 0.4 (0.3) , n=7 | 0.53 | -0.4 |  | 0.9 (0.6) , n=8 | 1.0 (0.8) , n=7 | 1.14 | 0.1 |  | 1.0 (0.9) , n=9 | 0.9 (0.4) , n=6 | 0.86 | -0.1 |
| *p < 0.05, Tanner Stage I-II compared to Tanner Stage III-V. | | | | | | | | | | | | | | |

| **Table S3-A-1**  **Linear Regression Analysis of Lung Function and ACQ6 by Sex Hormone Levels in Males 6-18 Years with Asthma and BMI >85 percentile**  **(n=23)** | | | | | |
| --- | --- | --- | --- | --- | --- |
| Outcome variable | Covariate remaining in the model* | Beta coefficient | Covariate p value | R squared | P value of the model |
| Pre-BD FEV_­1_  (%) | Log (DHEA-S) | 11.721 | 0.029 | 0.207 | 0.029 |
|  |  |  |  |  |  |
| Post-BD FEV_­1_­ (%) | Log (DHEA-S) | 12.285 | 0.0258 | 0.215 | 0.0258 |
|  | | | | | |
| Pre-BD FVC  (%) | log (DHEA-S) | 12.44 | 0.022 | 0.225 | 0.022 |
|  | | | | | |
| ACQ6 | Log (DHEA-S) | -0.674 | 0.003 | 0.35 | 0.003 |
|  |  |  |  |  |  |
|  |  |  |  |  |  |
| *Results reflect final results of a model that started with testosterone and DHEA-S. Covariates that were not significantly associated with the outcome in this model were then removed by backward selection and model was re-run with remaining variables. | | | | | |

| **Table S3-A-2**  **Linear Regression Analysis of Lung Function and ACQ6 by Sex Hormone Levels in Males 6-18 Years with Asthma and BMI <85 percentile**  **(n=22)** | | | | | |
| --- | --- | --- | --- | --- | --- |
| Outcome variable | Covariate remaining in the model* | Beta coefficient | Covariate p value | R squared | P value of the model |
| Pre-BD FEV_­1_  (%) | Log (DHEA-S) | 4.114 | 0.33 | 0.048 | 0.33 |
|  |  |  |  |  |  |
| Post-BD FEV_­1_­ (%) | Log (DHEA-S) | 5.55 | 0.197 | 0.081 | 0.197 |
|  | | | | | |
| Pre-BD FVC  (%) | log (DHEA-S) | 4.083 | 0.284 | 0.057 | 0.284 |
|  | | | | | |
| ACQ6 | Log (DHEA-S) | -0.272 | 0.356 | 0.043 | 0.356 |
|  |  |  |  |  |  |
|  |  |  |  |  |  |
| *Results reflect final results of a model that started with testosterone and DHEA-S. Covariates that were not significantly associated with the outcome in this model were then removed by backward selection and model was re-run with remaining variables. | | | | | |

| **Table S3-B-1**  **Multi-Variable Regression Analysis of Lung Function and ACQ6 by Sex Hormone Levels in Females 6-18 Years with Asthma and BMI >85 percentile**  **(n=13)** | | | | | |
| --- | --- | --- | --- | --- | --- |
| Outcome variable | Covariate remaining in the model* | Beta coefficient | Covariate p value | R squared | P value of the model |
| Pre-BD FEV_­1_  (%) | Estradiol | -0.065 | 0.637 | 0.021 | 0.637 |
|  |  |  |  |  |  |
| Post-BD FEV_­1_ ­(%) | Estradiol | 0.0347 | 0.749 | 0.01 | 0.749 |
|  | | | | | |
| Pre-BD FVC  (%) | Estradiol | -0.146 | 0.228 | 0.129 | 0.146 |
|  | | | | | |
| Post-BD FVC (%) | log (DHEA-S) | -5.907 | 0.055 | 0.295 | 0.055 |
|  | | | | | |
| ACQ6 | log (DHEA-S) | 0.541 | 0.228 | 0.129 | 0.228 |
| *Results reflect final results of a model that started with log (DHEA-S), estradiol, progesterone, and free testosterone as covariates. Covariates that were not significantly associated with the outcome in this model were then removed by backward selection and model was re-run with remaining variables. | | | | | |

| **Table S3-B-2**  **Multi-Variable Regression Analysis of Lung Function and ACQ6 by Sex Hormone Levels in Females 6-18 Years with Asthma and BMI <85 percentile**  **(n=12)** | | | | | |
| --- | --- | --- | --- | --- | --- |
| Outcome variable | Covariate remaining in the model* | Beta coefficient | Covariate p value | R squared | P value of the model |
| Pre-BD FEV_­1_  (%) | Estradiol | -0.479 | 0.12 | 0.2247 | 0.12 |
|  |  |  |  |  |  |
| Post-BD FEV_­1_ ­(%) | Estradiol | -0.4184 | 0.134 | 0.209 | 0.135 |
|  | | | | | |
| Pre-BD FVC  (%) | Estradiol | -0.291 | 0.276 | 0.117 | 0.276 |
|  | | | | | |
| Post-BD FVC (%) | Estradiol | -0.151 | 0.555 | 0.036 | 0.555 |
|  | | | | | |
| ACQ6 | log (DHEA-S) | 0.388 | 0.124 | 0.220 | 0.388 |
| *Results reflect final results of a model that started with log (DHEA-S), estradiol, progesterone, and free testosterone as covariates. Covariates that were not significantly associated with the outcome in this model were then removed by backward selection and model was re-run with remaining variables. | | | | | |

| **Table S2**  **Univariable Regression Analysis of ACQ6 by Age in children 6-18 Years with Asthma** | | | | | |
| --- | --- | --- | --- | --- | --- |
|  | | | | | |
| Outcome variable | Covariate remaining in the model* | Beta coefficient | Covariate p value | R squared | P value of the model |
| **Girls** | | | | | |
| **ACQ6** | Age | 0.055 | 0.447 | 0.025 | 0.447 |
| **ACQ6** | Age <12 vs. Age >12 (assuming age 12 defines puberty) | 0.397 | 0.394 | 0.032 | 0.394 |
| **ACQ6** | **Are you still menstruating (yes vs. No)** | 0.939 | 0.031 | 0.186 | 0.031 |
| **Boys** | | | | | |
| **ACQ6** | Age | -0.09 | 0.099 | 0.061 | 0.099 |

**Online Supplement Figures**

**
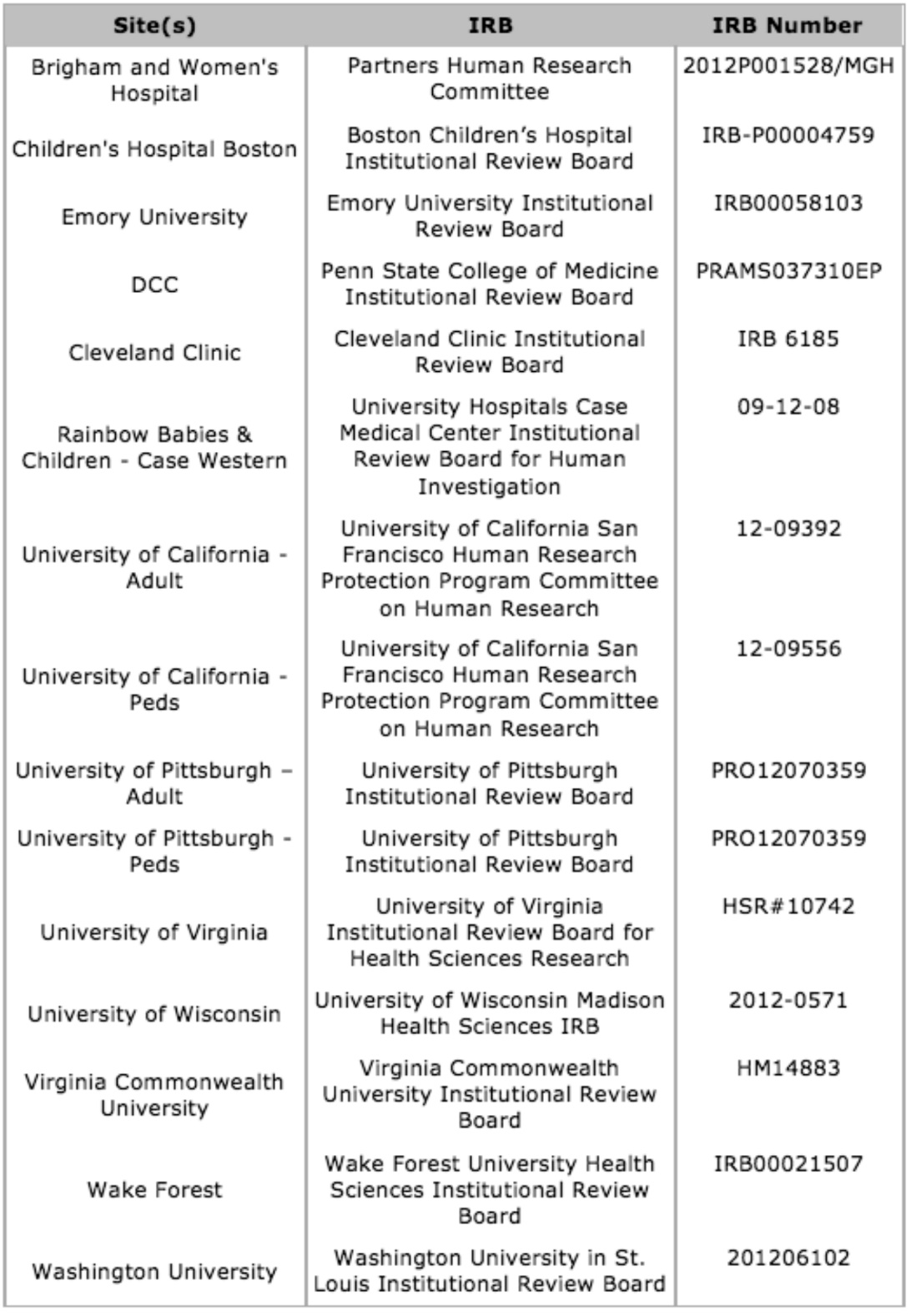
**

Supplementary Figure S1: Participating institutions, involved Institutional Review Boards (IRB) and IRB numbers.


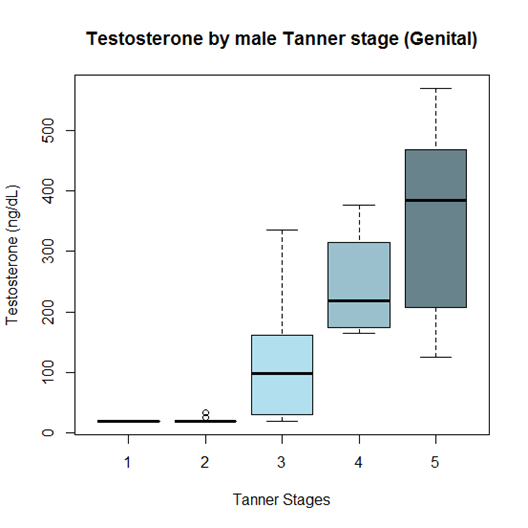


A.


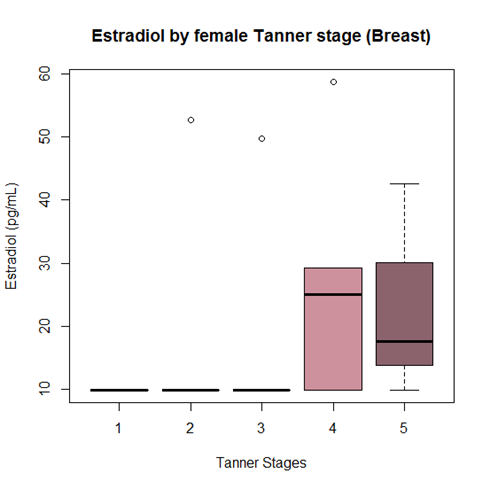


B.

Supplementary Figure S2: Sex hormone levels by Tanner stage for testosterone in males (A) and estradiol in females (B). Box plots are median and confidence intervals.


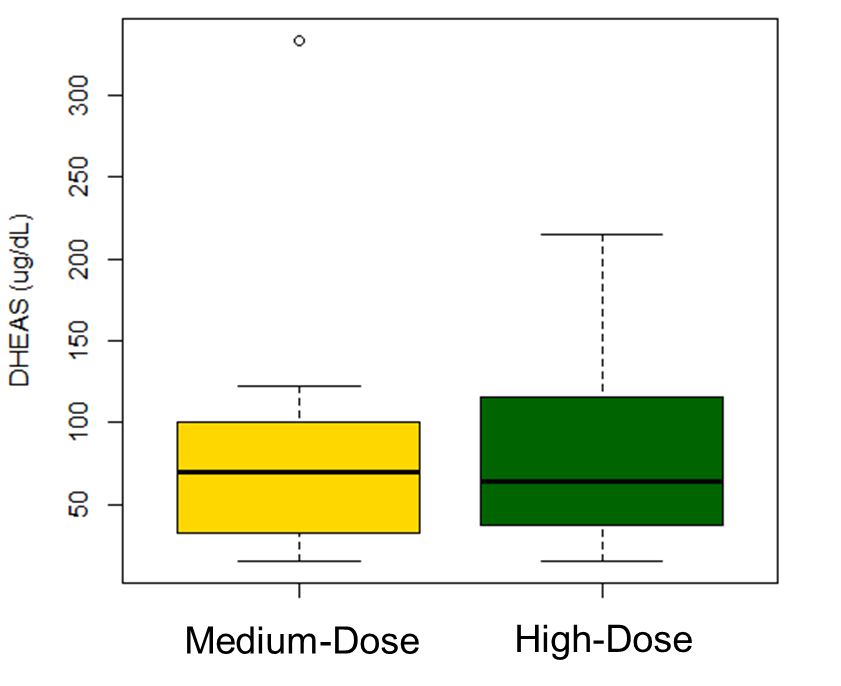


Supplementary Figure S3. DHEA-S levels in boys with asthma treated with medium-dose and high-dose inhaled corticosteroids. Box plots are median and confidence intervals.
